# The value of convolutional neural networks-based deep learning model in differential diagnosis of space-occupying brain diseases

**DOI:** 10.3389/fneur.2023.1107957

**Published:** 2023-02-02

**Authors:** Xiuling Miao, Tianyu Shao, Yaming Wang, Qingjun Wang, Jing Han, Xinnan Li, Yuxin Li, Chenjing Sun, Junhai Wen, Jianguo Liu

**Affiliations:** ^1^Department of Neurology, School of Medicine, South China University of Technology, Guangzhou, China; ^2^Department of Neurology, The Sixth Medical Center of PLA General Hospital of Beijing, Beijing, China; ^3^School of Life Science, Beijing Institute of Technology, Beijing, China; ^4^Department of Neurosurgery, Xuanwu Hospital, Capital Medical University, Beijing, China; ^5^Department of Radiology, The Sixth Medical Center of PLA General Hospital of Beijing, Beijing, China

**Keywords:** convolutional neural network, space-occupying brain lesions, diagnosis, differential, magnetic resonance imaging, tumefactive demyelinating lesions

## Abstract

**Objectives:**

It is still a challenge to differentiate space-occupying brain lesions such as tumefactive demyelinating lesions (TDLs), tumefactive primary angiitis of the central nervous system (TPACNS), primary central nervous system lymphoma (PCNSL), and brain gliomas. Convolutional neural networks (CNNs) have been used to analyze complex medical data and have proven transformative for image-based applications. It can quickly acquire diseases' radiographic features and correct doctors' diagnostic bias to improve diagnostic efficiency and accuracy. The study aimed to assess the value of CNN-based deep learning model in the differential diagnosis of space-occupying brain diseases on MRI.

**Methods:**

We retrospectively analyzed clinical and MRI data from 480 patients with TDLs (*n* = 116), TPACNS (*n* = 64), PCNSL (*n* = 150), and brain gliomas (*n* = 150). The patients were randomly assigned to training (*n* = 240), testing (*n* = 73), calibration (*n* = 96), and validation (*n* = 71) groups. And a CNN-implemented deep learning model guided by clinical experts was developed to identify the contrast-enhanced T_1_-weighted sequence lesions of these four diseases. We utilized accuracy, sensitivity, specificity, and area under the curve (AUC) to evaluate the performance of the CNN model. The model's performance was then compared to the neuroradiologists' diagnosis.

**Results:**

The CNN model had a total accuracy of 87% which was higher than senior neuroradiologists (74%), and the AUC of TDLs, PCNSL, TPACNS and gliomas were 0.92, 0.92, 0.89 and 0.88, respectively.

**Conclusion:**

The CNN model can accurately identify specific radiographic features of TDLs, TPACNS, PCNSL, and gliomas. It has the potential to be an effective auxiliary diagnostic tool in the clinic, assisting inexperienced clinicians in reducing diagnostic bias and improving diagnostic efficiency.

## 1. Introduction

Non-neoplastic space-occupying brain lesions with an atypical enhancement pattern, and/or associated mass effect such as tumefactive demyelinating lesions (TDLs) and tumefactive primary angiitis of the central nervous system (TPACNS) are often misdiagnosed with brain tumors in clinical practice, which are usually large (>2 cm) (1, 2). And the differential diagnosis before treatment is crucial because their treatment strategies and prognosis are markedly different (3). Some TDLs and TPACNS that are misdiagnosed as neoplasms suffer unnecessary surgical intervention or even radiation treatment, which may cause irreparable brain tissue damage or radiation encephalopathy with residual impairment. On the other hand, some brain tumors, such as gliomas and primary central nervous system lymphoma (PCNSL), which make up about 85% of all primary brain tumors and are the most common primary CNS malignant tumors (4), are misdiagnosed as TDLs or TPACNS because the pathology of the biopsy did not reveal tumor cells due to the early stage of the disease, the use of corticosteroids prior to the biopsy, or the failure to access the core of the lesion during the biopsy, resulting in delayed treatment (5, 6).

MRI is the most intuitive and non-invasive method for preoperative diagnosis of space-occupying brain diseases (7). However, due to uneven conditions of hospitals and clinicians' experience around the country, coupled with the fact that imaging differential diagnosis is somewhat subjective and lacks meticulous quantitative indicators, misdiagnosis is still common in clinical practice. Previous studies have reported that the diagnostic accuracy of skilled and experienced clinicians between PCNSL and gliomas ranges from 62.3 to 86.9% (8).

Deep learning, a subfield of machine learning, has been widely applied in recent years to develop an automated, semi-automatic, or hybrid model that can accurately and quickly classify and segment lesions in brain scans (9, 10). Imaging features are automatically acquired from data, then generated, organized into layers, and appropriately weighted on their own with high levels of complexity rather than needing researchers to identify and manually program certain characteristics (11, 12). Largely for this reason, deep learning techniques have increased state of the art classification accuracy by, sometimes, more than 30%, compared to the past decade's struggles to achieve gains of <2% (13). Convolutional neural network (CNN) is an important network structure in deep learning (14). It is well-known for its weight-sharing network structure, which reduces the network model's complexity and the number of weights to make it more resembling a biological neural network. In CNN, the picture can be utilized directly as the network's input, avoiding the complicated process of feature extraction and data reconstruction required by conventional recognition algorithms. CNN has also become the focus of research in the field of image recognition due to its remarkable invariance to image translation, scale, tilt, and other forms of deformation (15).

Combining the benefits of clinicians and CNN is anticipated to increase the classification accuracy of brain lesions (16). Thus, we put TDLs, TPACNS, PCNSL, and gliomas four space-occupying brain diseases with the most similar radiographic features together, and attempted to identify the four types of lesions by an MRI-based deep learning approach with specialist doctors' experience in a large data set. And we aimed to use this CNN model to improve diagnostic efficiency and accuracy of clinicians.

## 2. Materials and methods

### 2.1. Patients and image acquisitions

This study was approved by the ethical committee of our hospital (HZKY-PJ-2022-22). In total, 480 patients' data (116 TDLs, 64TPACNS, 150 PCNSL and 150 gliomas) from January 2010 to January 2021 were collected in our hospital. The whole data set was randomly separated into four sub-datasets for training, testing, calibration, and validation of the CNN model and the ratio of TDLs, TPACNS, PCNSL and gliomas was similar in each sub-dataset. Sub-dataset 1 (training set): 50% of the entire database cases were randomly chosen, comprising 58 TDLs, 32 TPACNS, 75 PCNSL, and 75 gliomas patients, for a total of 240 patients for preliminary learning of the CNN model. Sub-dataset 2 (testing set): 15% of the database's patients (17 TDLs, 10 TPACNS, 23 PCNSL, and 23 gliomas, for a total of 73 patients) were chosen at random as the testing set for the pre-trained model. Sub-dataset 3 (calibration set): randomly selected 20% of the overall database (23TDLs, 13 TPACNS, 30 PCNSL, and 30 gliomas, total of 96 patients), then selected targeted images as a calibration set based on test results to enhance its algorithm; Sub-dataset 4 (validation set): The remaining 15% of the total database (18 TDLs, 9 TPACNS, 22 PCNSL, and 22 gliomas, 71 patients in total) were used as the validation set of the improved CNN model.

The inclusion criteria were: (1) TDLs, TPACNS, PCNSL, or gliomas was proven by histopathology or diagnosed by the corresponding criteria of diseases (17–20); (2) underwent contrast-enhanced T1-weighted imaging (CE-T_1_WI). The exclusion criteria were: (1) missing clinical information; (2) no data on enhanced MRI; (3) MR images with obvious artifact.

### 2.2. MRI acquisition and lesions segmentation

The MRI was performed on 1.5T and 3T scanners including Verio (Siemens, Erlangen, Germany) or Signa (GE Healthcare, Milwaukee, Wisconsin), equipped with an eight-channel head coil. Dadopentetate dimeglumine was injected intravenously at doses based on the body weight of patients (0.1 mmol/kg), then the CE-T_1_WI images were obtained.

**Figure 1:**
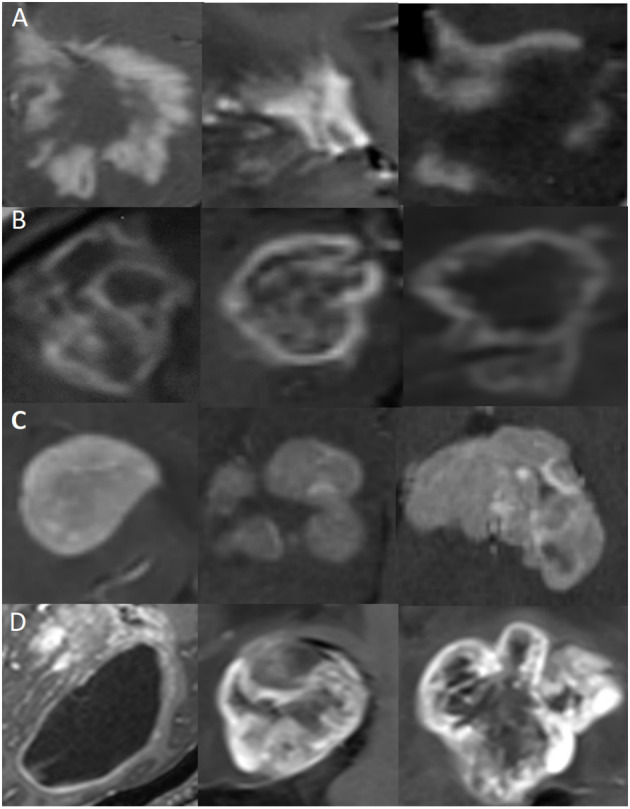
Representative MR images of clipped regions of interest (ROIs) for the four types of diseases. Each row represents a distinct disease, and each image is from a different patient. **(A)** ROIs from TDLs. **(B)** ROIs from TPACNS. **(C)** ROIs from PCNSL. **(D)** ROIs from gliomas.

The regions of interest (ROIs): ROIs of these four types of diseases were manually delineated on CE-T_1_WI (Figure 1). Then cropped ROIs in MR images and used the reduced images for the CNN model training and testing. Each patient was screened for 5–10 continuous slices with clear lesions by a neuroradiologist (with 15 years of experience) and a neurologist (with 10 years of experience) for computer model learning and testing.

### 2.3. Statistical analysis

All continuous variables were expressed as the mean ± standard deviation and the median, and categorical variables were expressed as the number (percentage), respectively. One-way analysis of variance (ANOVA) and Pearson's chi-square tests were used to compare the group differences with regard to patient age and sex ratio by SPSS (version 23.0, IBM Corporation, Armonk, NY, USA). *P* < 0.05 was considered statistically significant. The receiver operating characteristic (ROC) curve was used to show the area under the curve (AUC), and accuracy to evaluate the performance of the classification model. The accuracy was used as the main metric for comparisons of the CNN model and clinicians.

### 2.4. Algorithm Implementation

#### 2.4.1. Data pre-processing

In order to ensure the randomness of the data at each stage and the robustness of the algorithm, we randomly divided the whole data set into four sub-datasets in advance. And we cut the ROIs from MR images and trained the CNN model with cropped images to reduce the interference from the rest of the brain tissue. During the training phase, data enhancement operations such as rotation, folding, and cropping were done on each batch's data to achieve the effect of enlarging the training set *via* the OpenCV function library. Each image has a chance of 0.5 for each rotation angle, affine transformation, image clipping with a certain aspect ratio, and image folding. This process roughly doubles the quantity of data.

#### 2.4.2. CNN model development

CNN is widely utilized in data classification and has made significant progress because to its local area perception, sampling in space or time, and shared weight. However, CNN has some downsides as well. As the number of network layers increases, the identification impact first improves but then falls as the gradient either vanishes or extends. Thus, the residual block was introduced into CNN as an enhancement. The short-cut residual connection between continuous convolution layers eliminates the phenomenon of disappearing gradients and facilitates CNN training by permitting gradients to flow directly (21).

ResNet-18 is a deep convolutional network with residual blocks. And it has 18 layers with weight parameters, including 17 convolutional layers and one fully connected layer. In this study, all MR images are grayscale image, which reduces the training difficulty of the model and the demand for sample size. Therefore, we adopted ResNet-18 as the basic network for feature extraction of MR images and set the number of neurons in the last layer of the neural network to the number of categories to be classified (TDLs, TPACNS, PCNSL, and gliomas, respectively). The convolution layer parameters were obtained by loading the parameters of ResNet-18 that have been trained on the CIFAR-10 public dataset (22), and the neural network updated the parameters of the last layers by learning specific downstream tasks (https://github.com/shaitaiyangmie/train-for-classify).

Due to the limitation of the amount of medical imaging data, when training a supervised learning model, the training degree of the neural network model is often restricted and the performance of the model will be affected. Transfer learning is a novel approach to solving different but related issues by utilizing existing knowledge (23). Previous studies have demonstrated that transfer learning can lessen the necessity of the annotation procedure by reusing deep learning models trained on a different task and then refining them using data from the new task (24) and this method has been widely used in medical image problems by using a pre-trained model on large datasets, and then fine-tuning it with a small dataset. Therefore, we combined ResNet-18 with the transfer learning method, to improve the feature extraction ability of the network in limited datasets. Then, we used the training data collected for this study to fine-tune the original model's parameters and improve the algorithm's performance.

When we trained the neural network in the local GPU environment, the shape of input image was set to 64^*^64, the batch size was set to 64, the Epoch was set to 500, and the Learning Rate was set to 1e-4. After about 200 Epochs, when the accuracy no longer increases, the model can be approximately considered to have reached a state of convergence.

#### 2.4.3. Experts feedback to form a closed loop

**Figure 2:**
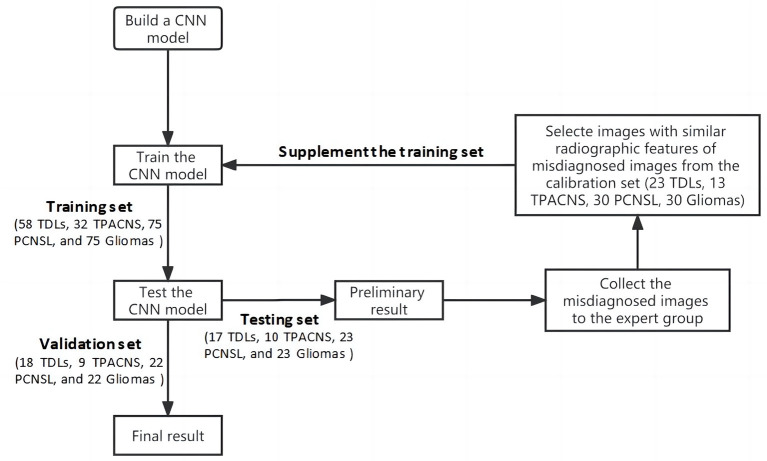
Flow chart of the training of the CNN model.

Compared with natural images, the amount of medical image data is relatively scarce, and as the radiographic heterogeneity of the above diseases is high, the image with similar characteristics are relatively insufficient, which increases the difficulty of training deep learning models. Thus, the misdiagnosed images of the CNN model in the first round of test was collected for the expert panel, which included a neurologist (with 18 years of experience), a neurosurgeon (with 20 years of experience), and a neuroradiologist (with 15 years of experience), to evaluate the weight of different radiographic characteristics of different diseases in the number. By adding images from calibration set with similar radiographic features of misdiagnosed images into the original training data, and train the model again on the basis of the original model parameters, to simulate the process of error correction of the expert guided the CNN model and form a closed-loop mechanism to improve the algorithm performance through continuous iteration (Figure 2).

#### 2.4.4. Human-computer interface development

**Figure 3:**
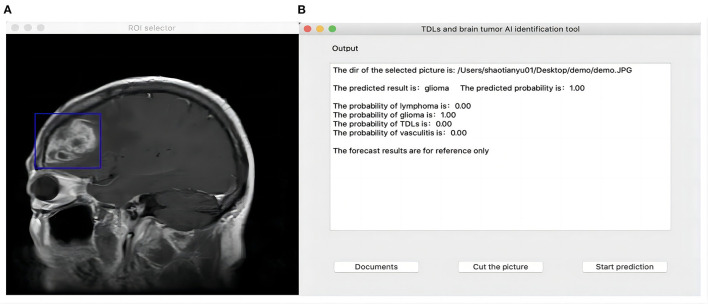
Human-computer interface. **(A)** ROI select interface. **(B)** Output interface.

In 2018, Zia et al. proposed a suitable size of moveable rectangular window to segmented and extract feature of brain neoplasm region which was used discrete wavelet transformed for feature extraction, principal component analysis for feature selection and support vector machine for classification (25). Referring to their concept, we developed a Python-based user interface and used the PyQt5 function library for functional expansion so that the CNN algorithm could be used in real clinical auxiliary diagnosis. Users can import the original image to be predicted into the interface, and when the image is presented on the interface, they can drag the mouse to select a rectangle region containing the lesion, then click “prediction” to identify it. While giving the classification results, the interface will also display the confidence level of the image picture for TDLs, TPACNS, PCNSL and gliomas, for reference (Figure 3). The script for interface building was available online (https://github.com/shaitaiyangmie/Brain-Imaging-Methods).

#### 2.4.5. Compare with neuroradiologists

Two blinded senior neuroradiologists with 15 years of experience independently identified these four categories of diseases in 71 cases from the validation set. Each neuroradiologist got access to the whole pictures produced by the various MRI scanners. The number of cases correctly diagnosed by two senior neuroradiologists was independently tallied, and their respective correct diagnosis rates were then computed. Finally, a comparison was made between the diagnostic precision of neuroradiologists and the CNN model.

## 3. Results

### 3.1. Subject' clinical characteristics

Pathological confirmation was obtained for 64 patients with TPACNS, 150 patients with PCNSL, and 150 patients with gliomas (including 38 WHO grade II, 54 grade III, and 58 grade IV gliomas), while 72 of 116 TDLs were pathologically validated and 44 were clinically identified (17). There were significant differences in age (*P* < 0.001) between patients with PCNSL and TDLs, TPACNS, gliomas, but no significant differences in gender (*P* = 0.661) (Table 1), consistent with previous reports (26, 27).

**Table 1:** Clinical characteristics of subjects.

	**TDLs**	**TPACNS**	**PCNSL**	**Gliomas**	***P*-Value**
Number of subjects	116	64	150	150	
Age (years)	37.29 ± 14.06	38.45 ± 14.08	57.60 ± 13.39	46.87 ± 16.14	<0.001
Gender					0.661
Male	55	35	90	83	
Famale	61	29	60	67	

The mean ± standard deviation of age is shown. The P-values are obtained from the comparison between TDLs, TPACNS, PCNSL, and gliomas. TDLs, tumefactive demyelinating lesions; TPACN, tumefactive primary angiitis of the central nervous system; PCNSL, primary central nervous system lymphoma.

According to clinical data, TDLs, PACNS, PCNSL, and gliomas are frequently misdiagnosed, and clinicians were predisposed to consider tumors in the initial diagnosis of space-occupying brain disorders. The median time between the onset of symptoms and the final diagnosis was 2 months (1–50 months) for TDLs, 2 months (0.50–72.00 months) for PACNS, 1 month (0.25–60.00 months) for PCNSL, and 2 months (0.50–144.00 months) for gliomas. Fifteen (10%) patients with PCNSL were misdiagnosed with inflammatory demyelinating disease, and six (4%) of those patients were remained misdiagnosed following the initial biopsy. Eventually, a second brain biopsy confirmed the diagnosis of PCNSL.

### 3.2. Diagnostic performance

**Figure 4:**
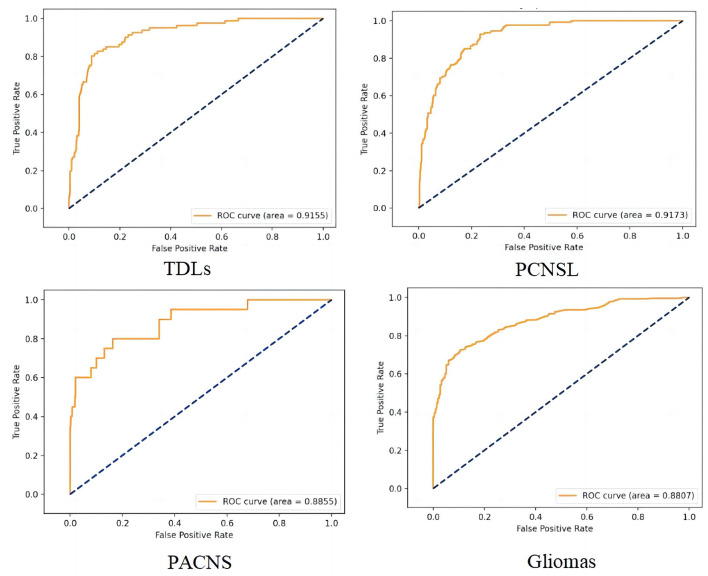
The receiver operating characteristic (ROC) curve of the four different diseases. False positive rate (FPR) = 1-specificity, True positive rate (TPR) = sensitivity.

We trained the CNN model with data from the training and calibration sets. And the model was basically stable after 500 epochs. Then we fed all of the data from the validation set into the CNN model for classification and achieved an overall accuracy of 87% with AUCs of 0.92, 0.92, 0.88, and 0.89 for TDLs, PCNSL, gliomas, and TPACNS, respectively (Figure 4). In addition, we separately ran a test for Glioblastoma (GBM), which obtained an AUC of 0.95 and the accuracy, sensitivity, and specificity are presented in Table 2. The diagnostic performances of the two senior neuroradiologists were 73 and 75%, respectively and the overall accuracy was 74%. In general, the CNN model's diagnostic accuracy was higher than that of senior neuroradiologists.

**Table 2:** Diagnostic performance of the CNN model and neuroradiologists.

**CNN model and neuroradiologists**		**ACC**	**SEN**	**SPE**
CNN Model	TDLs	0.88	0.78	0.96
	TPACNS	0.85	0.75	0.86
	PCNSL	0.89	0.93	0.89
	Gliomas	0.88	0.82	0.81
Neuroradiologists1	-	0.73	-	-
Neuroradiologists2	-	0.75	-	-

AUC, area under the curve; ACC, accuracy; SEN, sensitivity; SPE, specificity.

We also created a human-computer interaction interface to assist users in selecting the area of lesions. The interface will present the confidence level of the MR images for TDLs, TPACNS, PCNSL, and gliomas as a reference while providing classification results.

## 4. Discussion

In this work, we built a CNN-based differential diagnostic model to assist clinicians in differentiating TDLs, TPACNS, PCNSL, and gliomas from MRI. We chose ResNet-18 as the foundational model and combined it with transfer learning to achieve a more accurate understanding of the radiographic features of diseases on limited datasets. Previous research demonstrated that the CE-T_1_WI provided the best contrast for distinguishing space-occupying brain diseases and is widely utilized in conventional MR screening procedures for cerebral mass lesions (1, 28, 29). In order to acquire more data and achieve more accurate classification results, we trained the CNN model only with CE-T_1_WI images in this study.

Numerous studies have been conducted utilizing a variety of feature extraction/selection and classification algorithms to classify medical images, and most of them have shown satisfactory performance (29–32). As far as we are aware, no research has been done to date utilizing artificial intelligence technology to identify the above four types of space-occupying brain diseases. TDLs and TPACNS are the most commonly misdiagnosed space-occupying diseases due to lack of specificity in clinical presentation and the extensive heterogeneity on MRI. And most research focused on just two or three types of diseases with large and substantial lesions, such as GBM and PCNSL (28, 33, 34). GBM is a grade IV astrocytoma that is also the most prevalent and severe brain cancer (35), but is more readily distinguished from other space-occupying brain lesions than other kinds of gliomas due to its massive lesions and necrosis in the lesions. Low grade gliomas (LGG) and other high grade gliomas (HGG) (20), on the other hand, are harder to identify on MRI since their lesions are non-specific and often missed or confused with other diseases (36). Therefore, to be more applicable to the current clinical context with the complex etiology of space-occupying diseases, we included TDLs, TPACNS, PCNSL, LGG (diffuse astrocytoma, ependymoma), and HGG (GBM, anaplastic astrocytoma, anaplastic oligodendroglioma) patients in our research and attempted to differentiate these types of lesions at the same time.

In order to overcome the high radiographic heterogeneity and relative scarcity of image data with similar characteristics of the aforementioned diseases, we cropped ROIs in MR images and used the reduced images for CNN model training and testing to minimize interference from other brain tissues. And the expert group supplemented the training set with images that share similar radiographic characteristics with incorrectly diagnosed images of the CNN model to simulate the process of error correction by the expert-guided CNN model. Our CNN model finally achieved a total accuracy of 87%, which is better than that of the senior neuroradiologists (74%). In addition, we tested GBM images individually in our CNN model and had an AUC of 0.95.

According to our data, clinicians were more likely to consider tumors in the absence of dynamic imaging data. Although most patients were diagnosed 1–2 months after the onset of symptoms, the intervals ranged from 1 week to 80 months. It was due to the fact that space-occupying brain diseases are difficult to differentiate and most clinicians are inept at doing so, resulting in a wide range of diagnoses. Our CNN model may assist clinicians in swiftly acquiring space-occupying brain disease imaging characteristics and correcting their diagnostic bias. On the basis of clinical expertise and the CNN model's prediction findings, clinicians can establish a tentative diagnosis of a condition and then undertake the necessary tests to confirm the tentative diagnosis. When patients are suspected of having TDLs, testing with oligoclonal bands, aquaporin-4 antibody, glial fibrillary acidic protein antibody, and myelin oligodendrocyte glycoprotein antibody should be conducted (37). High-resolution wall MRI can help to diagnose TPACNS (18). And if a tumor is suspected, brain biopsies should be performed as soon as possible so that the pathological type can guide the further treatment or surgical excision (38). Moreover, pathological features of PCNSL with sentinel lesions or following steroids therapy may mimic TDLs (39). Thus, combination of clinical, neuroimaging pathological and follow-up information are essential for an accurate diagnosis.

### 4.1. Limitations

First, even though we randomly separated the whole data set into four sub-datasets to reduce it, the retrospective character of the research may have introduced some selection bias. Second, there were still some challenging situations that CNN models misdiagnosed. Since the appearance of tumor and non-neoplastic lesions changed as the disease progressed, and imaging performance during the early stages of diseases was often non-specific, which made diagnosis more challenging. In future research, we intend to stage the patient's imaging data according to the disease course and train the computer model's hierarchical classification to summarize the radiographic characteristics in various disease courses in order to enhance the model's performance. Thirdly, the proposed CNN model is still in its infancy and is only able to recognize CE-T_1_WI sequences. In the future, we will include patient information such as multi-parametric MRI sequences, age, and symptoms into the computer model's diagnosis to improve its accuracy.

## 5. Conclusion

It is essential to enhance the decision-making skills of doctors in neuroscience applications. The CNN model has an obvious advantage over clinicians in identifying particular radiographic features of TDLs, TPACNS, PCNSL, and gliomas on MR images. And that it might be utilized in the clinic as an additional diagnostic tool to assist inexperienced clinicians in minimizing diagnostic bias and improving the diagnostic efficiency and accuracy of space-occupying brain diseases.

## Data availability statement

The original contributions presented in the study are included in the article/supplementary material, further inquiries can be directed to the corresponding authors.

## Ethics statement

This study was approved by the Ethical Committee of the Sixth Medical Center of PLA General Hospital. Written informed consent for participation was not required for this study in accordance with the national legislation and the institutional requirements.

## Author contributions

JL, JW, and CS designed the original research and revised the paper. XM, TS, YW, QW, JH, XL, and YL conducted the research. XM, TS, and YW analyzed the data. XM and TS wrote the manuscript. All authors contributed to the article and approved the submitted version.

## References

[1] Suh CH Kim HS Jung SC Choi CG Kim SJ. MRI findings in tumefactive demyelinating lesions: a systematic review and meta-analysis. AJNR Am J Neuroradiol. (2018) 39:1643–9. 10.3174/ajnr.A5775 30115676 PMC7655270

[2] de Boysson H Boulouis G Dequatre N Godard S Neel A Arquizan C. Tumor-like presentation of primary angiitis of the central nervous system. Stroke. (2016) 47:2401–4. 10.1161/STROKEAHA.116.013917 27470990

[3] Paoletti M Muzic SI Marchetti F Farina LM Bastianello S Pichiecchio A. Differential imaging of atypical demyelinating lesions of the central nervous system. Radiol Med. (2021) 126:827–42. 10.1007/s11547-021-01334-y 33486703

[4] Ostrom QT Patil N Cioffi G Waite K Kruchko C Barnholtz-Sloan JS. Statistical report: primary brain and other central nervous system tumors diagnosed in the United States in 2013-2017. Neuro Oncol. (2020) 22:v1–96. 10.1093/neuonc/noaa200 33123732 PMC7596247

[5] Ng S Butzkueven H Kalnins R Rowe C. Prolonged interval between sentinel pseudotumoral demyelination and development of primary CNS lymphoma. J Clin Neurosci. (2007) 14:1126–9. 10.1016/j.jocn.2006.05.003 17890092

[6] Bajagain M Oyoshi T Hanada T Higa N Hiraki T Kamimura K. Histopathological variation in the demyelinating sentinel lesion of primary central nervous system lymphoma. Surg Neurol Int. (2020) 11:342. 10.25259/SNI_531_2020 33194276 PMC7655992

[7] Faehndrich J Weidauer S Pilatus U Oszvald A Zanella FE Hattingen E. Neuroradiological viewpoint on the diagnostics of space-occupying brain lesions. Clin Neuroradiol. (2011) 21:123–39. 10.1007/s00062-011-0073-6 21538040

[8] Nguyen AV Blears EE Ross E Lall RR Ortega-Barnett J. Machine learning applications for the differentiation of primary central nervous system lymphoma from glioblastoma on imaging: a systematic review and meta-analysis. Neurosurg Focus. (2018) 45:E5. 10.3171/2018.8.FOCUS18325 30453459

[9] Nazir M Shakil S Khurshid K. Role of deep learning in brain tumor detection and classification (2015 to 2020): a review. Comput Med Imaging Graph. (2021) 91:101940. 10.1016/j.compmedimag.2021.101940 34293621

[10] Zaharchuk G Gong E Wintermark M Rubin D Langlotz CP. Deep learning in neuroradiology. AJNR Am J Neuroradiol. (2018) 39:1776–84. 10.3174/ajnr.A5543 29419402 PMC7410723

[11] Akkus Z Galimzianova A Hoogi A Rubin DL Erickson BJ. Deep learning for brain MRI segmentation: state of the art and future directions. J Digit Imaging. (2017) 30:449–59. 10.1007/s10278-017-9983-4 28577131 PMC5537095

[12] Magadza T Viriri S. Deep learning for brain tumor segmentation: a survey of state-of-the-art. J Imaging. (2021) 7:19. 10.3390/jimaging7020019 34460618 PMC8321266

[13] Krizhevsky A Sutskever I Hinton G. ImageNet classification with deep convolutional neural networks. Adv Neural Inform Process Syst. (2012) 25:84–90. 10.1145/3065386

[14] LeCun Y Bengio Y Hinton G. Deep learning. Nature. (2015) 521:436–44. 10.1038/nature14539 26017442

[15] Yaqub M Jinchao F Zia MS Arshid K Jia K Rehman ZU. State-of-the-art CNN optimizer for brain tumor segmentation in magnetic resonance images. Brain Sci. (2020) 10:427. 10.3390/brainsci10070427 32635409 PMC7407771

[16] Aneja S Chang E Omuro A. Applications of artificial intelligence in neuro-oncology. Curr Opin Neurol. (2019) 32:850–6. 10.1097/WCO.0000000000000761 31609739

[17] Chinese guidelines for the diagnosis and management of tumefactive demyelinating lesions of central nervous system. Chin Med J. (2017) 130:1838–50. 10.4103/0366-6999.211547 28748858 PMC5547837

[18] Birnbaum J Hellmann DB. Primary angiitis of the central nervous system. Arch Neurol. (2009) 66:704–9. 10.1001/archneurol.2009.76 19506130

[19] Hoang-Xuan K Bessell E Bromberg J Hottinger AF Preusser M Ruda R. Diagnosis and treatment of primary CNS lymphoma in immunocompetent patients: guidelines from the European association for neuro-oncology. Lancet Oncol. (2015) 16:e322–32. 10.1016/S1470-2045(15)00076-5 26149884

[20] Louis DN Ohgaki H Wiestler OD Cavenee WK Burger PC Jouvet A. The 2007 WHO classification of tumours of the central nervous system. Acta Neuropathol. (2007) 114:97–109. 10.1007/s00401-007-0243-4 17618441 PMC1929165

[21] He KM Zhang XY Ren SQ Sun J. Deep residual learning for image recognition. In: 2016 IEEE Conference on Computer Vision and Pattern Recognition (CVPR). IEEE (2016). p. 770–8. 10.1109/CVPR.2016.90

[22] Krizhevsky A Hinton G. Learning multiple layers of features from tiny images. In: Handbook of Systemic Autoimmune Diseases, Vol. 1 (2009). p. 1–60. Available online at: https://www.researchgate.net/publication/306218037_Learning_multiple_layers_of_features_from_tiny_images33561989

[23] Image classification of fine-grained fashion image based on style using pre-trained convolutional neural network. In: 2018 IEEE 3rd International Conference on Big Data Analysis (ICBDA) (2018). p. 387–90.

[24] Kim J Yang E. Sea Fog identification from GOCI images using CNN transfer learning models. Electron. Switz. (2020) 9:311. 10.3390/electronics9020311

[25] Zia R Akhtar P Aziz A. A new rectangular window based image cropping method for generalization of brain neoplasm classification systems. Int J Imag Syst Tech. (2018) 28:153–62. 10.1002/ima.22266

[26] Grommes C DeAngelis LM. Primary CNS lymphoma. J Clin Oncol. (2017) 35:2410–8. 10.1200/JCO.2017.72.7602 28640701 PMC5516483

[27] Nakayama M Naganawa S Ouyang M Jones KA Kim J Capizzano AA. A review of clinical and imaging findings in tumefactive demyelination. AJR Am J Roentgenol. (2021) 19:1–12. 10.2214/AJR.20.23226 34010036

[28] Suh CH Kim HS Jung SC Park JE Choi CG Kim SJ. as a diagnostic biomarker for differentiating primary central nervous system lymphoma from glioblastoma: a systematic review and meta-analysis. J MAGN Reson Imaging. (2019) 50:560–72. 10.1002/jmri.26602 30637843

[29] Xia W Hu B Li H Shi W Tang Y Yu Y. Deep learning for automatic differential diagnosis of primary central nervous system lymphoma and glioblastoma: multi-parametric magnetic resonance imaging based convolutional neural network model. J Magn Reson Imaging. (2021) 54:880–7. 10.1002/jmri.27592 33694250

[30] Chang P Grinband J Weinberg BD Bardis M Khy M Cadena G. Deep-learning convolutional neural networks accurately classify genetic mutations in gliomas. AJNR Am J Neuroradiol. (2018) 39:1201–7. 10.3174/ajnr.A5667 29748206 PMC6880932

[31] Zhang Y Liang K He J Ma H Chen H Zheng F. Deep learning with data enhancement for the differentiation of solitary and multiple cerebral glioblastoma, lymphoma, and tumefactive demyelinating lesion. Front Oncol. (2021) 11:665891. 10.3389/fonc.2021.665891 34490082 PMC8416477

[32] Zhang M Young GS Chen H Li J Qin L McFaline-Figueroa JR. Deep*-*Learning detection of cancer metastases to the brain on MRI. J Magn Reson Imaging. (2020) 52:1227–36. 10.1002/jmri.27129 32167652 PMC7487020

[33] Yang Z Feng P Wen T Wan M Hong X. Differentiation of glioblastoma and lymphoma using feature extraction and support vector machine. CNS Neurol Disord Drug Targets. (2017) 16:160. 10.2174/1871527315666161018122909 27758687

[34] Kunimatsu A Kunimatsu N Yasaka K Akai H Kamiya K Watadani T. Machine learning-based texture analysis of contrast-enhanced MR imaging to differentiate between glioblastoma and primary central nervous system lymphoma. Magn Reson Med Sci. (2019) 18:44–52. 10.2463/mrms.mp.2017-0178 29769456 PMC6326763

[35] Grabowski MM Recinos PF Nowacki AS Schroeder JL Angelov L Barnett GH. Residual tumor volume versus extent of resection: predictors of survival after surgery for glioblastoma. J Neurosurg. (2014) 121:1115–23. 10.3171/2014.7.JNS132449 25192475

[36] Ryu YJ Choi SH Park SJ Yun TJ Kim JH Sohn CH. Glioma: application of whole-tumor texture analysis of diffusion-weighted imaging for the evaluation of tumor heterogeneity. PLoS ONE. (2014) 9:e108335. 10.1371/journal.pone.0108335 25268588 PMC4182447

[37] Hardy TA. Pseudotumoral demyelinating lesions: diagnostic approach and long-term outcome. Curr Opin Neurol. (2019) 32:467–74. 10.1097/WCO.0000000000000683 30844860

[38] Jiang T Mao Y Ma W Mao Q You Y Yang X. CGCG clinical practice guidelines for the management of adult diffuse gliomas. Cancer Lett. (2016) 375:263–73. 10.1016/j.canlet.2016.01.024 26966000

[39] Kvarta MD Sharma D Castellani RJ Morales RE Reich SG Kimball AS. Demyelination as a harbinger of lymphoma: a case report and review of primary central nervous system lymphoma preceded by multifocal sentinel demyelination. BMC Neurol. (2016) 16:72. 10.1186/s12883-016-0596-1 27206499 PMC4875602

